# Impact of COVID-19 on Cancer Patients: An Experience From a Tertiary Care Center in Northeast India

**DOI:** 10.7759/cureus.52247

**Published:** 2024-01-14

**Authors:** Sumit Kumar, Binoy Singh, Biswajit Dey, Vikas K Jagtap, Shalini Verma, Anthialisha Nongkynrih

**Affiliations:** 1 Radiation Oncology, North Eastern Indira Gandhi Regional Institute of Health and Medical Sciences, Shillong, IND; 2 Neurosurgery, All India Institute of Medical Sciences, Raipur, Raipur, IND; 3 Pathology and Laboratory Medicine, North Eastern Indira Gandhi Regional Institute of Health and Medical Sciences, Shillong, IND; 4 Medical Oncology, Kasturba Medical College, Manipal, Manipal, IND

**Keywords:** covid-19, cancer care, pandemic, health impact, cancer

## Abstract

Introduction: The COVID-19 pandemic affected the healthcare system worldwide. Cancer patients and oncologists faced challenges equally in the context of the pandemic. The present study was undertaken to assess the impact of COVID-19 on cancer patients, encompassing infection source, care type, treatment delays, and infection outcomes.

Materials and method: This single-center retrospective study was conducted between March 2020 and January 2022 at North Eastern Indira Gandhi Regional Institute of Health and Medical Sciences, Shillong, India. It examined COVID-19 cases in cancer patients with positive severe acute respiratory syndrome coronavirus 2 (SARS-CoV-2) reverse transcription-polymerase chain reaction (RT-PCR) results. Data collection included demographics, clinical details, COVID-19 specifics, treatment delays, and infection outcomes.

Result: In our study of 9,854 oncology patients' visits, 26 (0.26%) tested COVID-19 positive by RT-PCR, aged three to 70 years with a male-female ratio of 1:1.67. Twenty-three percent had comorbidities, mainly hypertension. Gastrointestinal cancers (30.8%) and hepatobiliary origin (15.5%) were common. Most patients (69.2%) had stage IV cancer, and 34.6% aimed for curative treatment. The majority of the patients (76.9%) were community-acquired, and the rest (23.1%) contracted during hospital stay. Fever (34.5%) and asymptomatic infection (30.8%) were common presentations. Six (23.1%) comorbid patients required ICU care. Median treatment delay was three weeks, with one COVID-19-related death (3.8%) and six cancer-related deaths. On follow-up, 19.2% had stable disease, 7.7% partial response, 7.7% recurrence, and 23.1% had progression.

Conclusion: Amid the pandemic, cancer patients safely received treatment. Mild cases were managed at home. Poor outcome was found in comorbid, severe COVID-19 cancer patients. However, the impact of treatment delays on long-term oncological outcomes needs further study.

## Introduction

The severe acute respiratory syndrome coronavirus 2 (SARS-CoV-2) that triggered the COVID-19 pandemic has caused extensive mortality and exposed the shortcomings of healthcare systems around the world. With restrictions or lockdowns of varying severity imposed, cancer care has been affected with some cancer centers being partially or completely converted to COVID-19 treatment facilities. Due to these factors, the COVID-19 pandemic has been a huge concern for oncologists and cancer patients [1,2].

While some reports suggest that active cancer, immunosuppressive treatments, and immunotherapy increased patients’ risk of developing severe COVID-19 infection, the risks from COVID-19 infection have to be weighed against the personalized risks of disease progression without treatment [1,2]. Thus, the National Institute of Clinical Excellence, the European Society for Medical Oncology, and other expert committees have all produced recommendations to address cancer patients' consideration of risk versus benefit of treatment during a pandemic by prioritizing and categorizing people [1,3].

## Materials and methods

This is a single-center, observational, retrospective study conducted at North Eastern Indira Gandhi Regional Institute of Health and Medical Sciences, Shillong, India. We collected data on all cancer patients diagnosed with COVID-19 between March 1, 2020 to January 30, 2022. Every patient underwent a clinical evaluation for COVID-19 in the screening area as per our institutional policy. Any patients who had an influenza-like illness (ILI) or severe acute respiratory illness (SARI) were subjected to reverse transcription-polymerase chain reaction (RT-PCR) for SARS-CoV-2. In addition, any patients who developed ILI or SARI during their hospital stay were also subjected to RT-PCR for SARS-CoV-2. Various Indian Council of Medical Research (ICMR)-approved kits were used during the pandemic. The SARS-CoV-2 specific genes that were amplified included the E gene, ORF 1ab, N gene, and RdRp gene.

All active cancer patients, who had a positive nasopharyngeal swab for SARS-CoV-2 by RT-PCR, were included in the study. These patients were either attending for day care chemotherapy or admitted for chemotherapy. Active cancer patients, who were positive for SARS-CoV-2 but had chemotherapy within the last four weeks, were also included in the study. All duplicate samples of patients, who tested positive within a three-month period from testing positive, were excluded as they fell into the Centers for Disease Control and Prevention (CDC) case definition of an existing case.

Data were collected through a review of the files of the patients, who attended or were admitted in the oncology department. Demographic data included age and gender. Clinical data included the site of malignancy, the Eastern Cooperative Oncology Group (ECOG) performance status, body mass index (BMI), stage, and treatment intent. Data specific to COVID-19 included source of COVID-19 infection, COVID-19 status, type of COVID-19 care, and delay in treatment due to COVID-19. Outcomes of the COVID-19 infection in these patients were collected. The last follow-up of these patients in the oncology department was also noted.

## Results

During this period, a total of 9,854 patients' visits were registered in the oncology outpatient department or were admitted to the oncology ward. Out of the total visits, 26 (0.26%) cases were COVID-19-positive confirmed on RT-PCR. The age of the patients ranged from three years to 70 years with a median age of 48 years. There were 12 (46.2%) males and 14 (53.8%) females with a male:female ratio of 1:1.67. Out of 26 patients, six patients (23.1%) had comorbidities. All six patients had hypertension, and two patients had both hypertension and diabetes mellitus. Thirteen patients had an ECOG performance status of 1 while 11 patients had an ECOG status of 2, and only two patients had ECOG status ≥3 (Table 1).

**Table 1:** Characteristics of the cancer patients with COVID-19 ECOG: Eastern Cooperative Oncology Group; BMI: body mass index

Patient characteristic		Number of patients	Percentage of patients
Age	<20	2	7.7
	≥20-<40	7	26.9
	≥40-<60	12	46.2
	≥60	5	19.2
Sex	Female	14	53.8
	Male	12	46.2
Comorbidities	Yes	6	23.1
	No	20	76.9
ECOG	1	13	50
	2	11	42.3
	≥ 3	2	7.7
BMI	<30	23	88.5
	≥30	3	11.5
Cancer site	Gastrointestinal	8	30.8
	Hepatobiliary	4	15.5
	Female genital tract	3	11.6
	Bone and soft tissue	2	7.7
	Head and neck	2	7.7
	Central nervous system	2	7.7
	Testis	1	3.8
	Skin	1	3.8
	Breast	1	3.8
	Lymph node	1	3.8
	Kidney	1	3.8
Stage	II	4	15.4
	III	4	15.4
	IV	18	69.2
Metastases	Multiple	3	11.6
	Single	14	53.8
	No metastasis	9	34.6
Treatment intent	Curative	9	34.6
	Palliative	17	65.4

Eight (30.8%) patients had primary gastrointestinal cancer, followed by four (15.5%) patients with a hepatobiliary origin. Advanced diseases were predominant, with 18 (69.2%) patients having stage IV cancer. Nine (34.6%) patients were planned for curative treatment. There were two main clusters of patients: six (23.1%) patients who developed COVID-19 while undergoing antitumor therapy in hospitals and 20 (76.9%) patients in their communities. All six hospitalized patients were shifted to the COVID ward, while another six (23.1%) patients, who had pre-existing comorbidities, required admission to the intensive care unit (ICU).

The most common presentation of COVID-19 infection was fever (n = 9, 34.5%), followed by asymptomatic COVID-19 infection (n = 8, 30.8%). Two patients had radiological evidence of COVID-19 pneumonia with symptoms of fever, cough, and shortness of breath. Twenty-two patients had anemia (84.6%), eight patients had leukopenia (30.8%), and 21 patients had lymphopenia (80.8%) at the time of diagnosis of COVID-19. Four patients (15.4%) had prolonged prothrombin time and elevated D-dimer levels.

The median duration of the treatment delay was three weeks. Only one (3.8%) died of COVID-19 pneumonia with ECOG status ≥3 and deranged coagulation profile. Another six patients died during follow-up due to complications related to the cancer. On the last follow-up of the rest of the patients, five (19.2%) patients had stable disease, two (7.7%) had partial response, two (7.7%) had recurrent disease, and six (23.1%) had progressive disease (Table 2).

**Table 2:** Impact of COVID-19 and follow-up of the patients

COVID-19 and follow-up of patients		Number of patients	Percentage of patients
Source of COVID-19 infection	Community	20	76.9
	Nosocomial	6	23.1
COVID symptoms	Asymptomatic	8	30.8
	Fever	9	34.6
	Cough	7	26.9
	Shortness of breath	2	7.7
	Throat pain	2	7.7
	Loss of appetite	1	3.8
	Bodyache	2	7.7
COVID care	Home isolation	14	53.8
	COVID ward	6	23.1
	COVID ICU	6	23.1
COVID-19 outcome	Recovered	25	96.2
	Expired	1	3.8
Status at the last follow-up	No evidence of disease	4	15.4
	Partial response	2	7.7
	Stable disease	5	19.2
	Progressive disease	6	23.1
	Recurrent	2	7.7
	Expired due to COVID-19	1	3.8
	Expired due to cancer	6	23.1

## Discussion

During the COVID-19 pandemic, there have been multiple reports of delays in cancer care due to overburdening in healthcare centers and COVID-related restrictions worldwide. There had been a considerable decrease in outpatient visits, oncological surgeries, outpatient chemotherapy, and even hospitalization during the pandemic, which were attributed to various reasons, such as travel restrictions, patient concerns, regulatory guidance, and sequestering of oncology staff [4]. Although there was a similar decrease in outpatient visits and hospitalization in our center, routine hospital services including cancer care continued during the COVID pandemic [5]. 

In the present study, around 0.26% of cancer patients had COVID-19, which is lower than the report by Liang et al. [6]. According to their findings, 1% of COVID-19 cases had a history of malignancy [6]. Lung cancer was listed as the most common cancer type by Liang et al. and Zhang et al. [6,7]. Meanwhile, in the present study, gastrointestinal cancer was the most frequent type. This difference could be attributed to the epidemiological factors related to cancer prevalence. The most common presentation in the present cohort was fever, followed by cough, which is similar to other studies [7]. Zhang et al. also reported eight (28.6%) cases of hospital-associated transmission in their cohort [7]. In the present cohort, six (23.1%) patients were suspected to have hospital-associated transmission, and all of them were provided hospital care in the COVID ward. Another six patients, who had additional comorbidities like hypertension and/or diabetes mellitus, were provided care in the ICU. The majority of the patients (53.8%) did not require hospitalization as they had mild infections.

A treatment interruption, a pause in their scheduled radiation treatment, or a delay in the start of the treatment were all considered delays in treatment [8]. The median delay in treatment was three weeks in the present cohort. A similar finding was reported by Mitra et al., who reported a median treatment delay of 2.6 weeks [8].

In the present study, there was only one mortality (3.8%) due to COVID-19 pneumonia and a deranged coagulation profile. Radiological evidence of COVID-19-related pulmonary changes and laboratory hematological parameters are known to influence outcomes in COVID-19 [7,9]. While some studies have reported a higher mortality due to COVID-19 infection in cancer patients, others have reported no association of COVID-19 with mortality [10,11]. A deranged coagulation profile, especially an increased level of D-dimer and prolongation of prothrombin time, has been associated with poor outcomes among COVID-19 patients [12]. Poor ECOG status is associated with poor outcome in cancer patients with COVID-19, whereas, increased BMI is not a determinant of poor outcome in this group of patients [13,14]. On the follow-up of the rest of the patients, six patients had progressive disease, two had recurrence, and six had died due to cancer-related complications. These findings raise the question of whether treatment delay affects the oncological outcome of these patients in the long run. An analysis of a larger cohort involving multiple centers and longer follow-ups of these patients is required to answer this question.

Thus, the limitations of the study include a smaller cohort with short-term follow-up. Moreover, treatment protocol and prognosis differ for different malignancies and different stages, which need consideration.

## Conclusions

This single-center retrospective study demonstrated that even amid the COVID-19 pandemic, patients could safely receive systemic anti-cancer treatment without any added risk of infection or mortality. The majority of cancer patients with mild symptoms were managed with home isolation and did not require further hospitalization. Factors, such as additional comorbidities, severity of COVID-19, and deranged laboratory parameters, were associated with poor outcomes. Whether the treatment delay affects the patients' long-term oncological outcome remains to be answered.
